# MiR-449b-5p targets lncRNA PSMG3-AS1 to suppress cancer cell proliferation in lung adenocarcinoma

**DOI:** 10.1186/s12890-020-01189-5

**Published:** 2020-05-29

**Authors:** Na Yue, Ming Ye, Ran Zhang, Yunquan Guo

**Affiliations:** Department of Pathology, the 3rd Affiliated Teaching Hospital of Xinjiang Medical University (Affiliated Cancer Hospital), NO.789 East Suzhou Street, New Urban Area, Urumqi, 830000 Xinjiang China

**Keywords:** Lung adenocarcinoma, PSMG3-AS1, miR-449b-5p, Prognosis

## Abstract

**Background:**

PSMG3-AS1 has been characterized as an oncogenic lncRNA in breast cancer, while its role in other cancers is unknown. This study investigated the role of PSMG3-AS1 in lung adenocarcinoma (LUAD).

**Methods:**

This study included 64 LUAD patients (42 males and 22 females) who were enrolled between May 2012 and May 2014. RT-qPCR was used to evaluate the expression levels of lncRNA. Cell proliferation analysis was performed using CCK-8 kit.

**Results:**

We found that upregulation of PSMG3-AS1 in LUAD predicted the poor survival of patients. MiR-449b-5p is downregulated in LUAD and the expression levels of LUAD were inversely correlated with the expression levels of PSMG3-AS1. MiR-449b-5p was predicted to target PSMG3-AS1, and overexpression of miR-449b-5p resulted in the downregulation of PSMG3-AS1 in LUAD cells. Cell proliferation analysis showed that overexpression of PSMG3-AS1 resulted in increased rate of cell proliferation. Overexpression of miR-449b-5p reduced the enhancing effects of PSMG3-AS1 on cell proliferation.

**Conclusions:**

Therefore, miR-449b-5p may target PSMG3-AS1 in LUAD to suppress cancer cell proliferation.

## Background

Lung cancer is considered as the leading cause of cancer-related mortality for decades worldwide [1]. The latest GLOBOCAN statistics reported that lung cancer caused 1,761,007 deaths, accounting for 18.4% of all cancer deaths in 2018 [2]. In the same year, there were 2,093,876 new cases of lung cancer, accounting for 11.6% of all new cancer cases [2]. It is estimated that more than 50% of lung cancer patients diagnosed with a localized tumor can live longer than 5 years, but only 16% of lung cancer patients are diagnosed at early stages [3]. Once distant metastasis occurs, only 6% of lung cancer patients can survive for 5 years [4]. Tobacco smoking is responsible for the majority of lung cancer cases, while this disease also affects never-smokers, indicating the complicated pathogenesis [5, 6]. Studies on the molecular mechanisms of lung cancer have identified critical molecular pathways involved in the development and progression of this disease [7, 8]. Characterization of lncRNAs involved in lung cancer provides novel insights into the development of targeted therapies [9, 10]. Non-coding RNAs (ncRNAs), such as long (> 200 nt) ncRNAs (lncRNAs) [11] and miRNAs [12] are critical players in cancer biology and either promote or suppress cancer development by regulating the expression of cancer-related genes. Therefore, regulating the expression of cancer-related ncRNAs may benefit cancer treatment. However, the functions of most ncRNAs in cancer remain unclear. PSMG3-AS1 was recently characterized as an oncogenic lncRNA in breast cancer [13], while its role in lung cancer remains unclear. Our bioinformatics analysis showed that PSMG3-AS1 may be targeted by miR-449b-5p, which is a tumor suppressive miRNA [14]. This study was therefore carried out to investigate the interaction between miR-449b-5p and PSMG3-AS1 in lung adenocarcinoma, a major subtype of lung cancer.

## Methods

### LUAD patients and tissue samples

This study was approved by the Ethics Committee of the 3rd Affiliated Teaching Hospital of Xinjiang Medical University. This study included 64 LUAD patients (42 males and 22 females) who were enrolled at the aforementioned hospital between May 2012 and May 2014. All LUAD patients were newly diagnosed cases and cases with other severe clinical disorders, such as other malignancies and chronic diseases, were excluded. No therapy was initiated before this study. The age of patients ranged from 42 to 66 years old, with a mean age of 54.2 ± 6.7 years old. Among the 64 patients, 51 were smokers or had a history of smoking. All patients signed the written informed consent. Paired LUAD and non-tumor tissues were collected from each patient through fine needle aspiration (FNA). All tissue specimens were confirmed by histopathological exams. All tissue samples were stored in liquid nitrogen before use.

### A 5-year follow-up

The 64 patients were staged according to AJCC criteria (8th edition). There were 8, 11, 21, and 24 cases of stage I, stage II, stage III and stage IV, respectively. Based on clinical stage, patients were either treated with chemotherapy, surgical resection, radiotherapy, targeted therapy, or the combination of these treatments. All patients were followed up for 5 years after admission to record their survival. All patients completed the follow-up.

### LUAD cells and transfections

LUAD cell lines H522 and H23 cells obtained from ATCC were used. Cell culture medium was composed of 10% FBS and 90% RPMI-1640 medium. Cell culture conditions were 5% CO_2_, 37 °C and 95% humidity. PSMG3-AS1 expression vector was constructed using pcDNA3.1 vector (Sigma-Aldrich) as backbone. Negative control (NC) miRNA (5′-CCGUAGGUGCACGUGAAACGAC-3′) and miR-449b-5p mimic (5′-AGGCAGUGUAUUGUUAGCUGGC-3′) were also purchased from Sigma-Aldrich. PSMG3-AS1 siRNA (5′-CUGUGCGUUCGUUCUGCUUC-3′) and NC siRNA (5′-GUCGUAGUUACCUUUGACGAU-3′) were purchased from Invitrogen. H522 and H23 cells were harvested at about 85% confluence. Lipofectamine 2000 (Invitrogen) was used to transfect the cells with 45 nM siRNA or 10 nM expression vector. Untransfected cells were used as the Control (C) cells, and NC miRNA- or empty vector-infected cells were used as NC cells. Cells were harvested 48 h later to perform the subsequent experiments.

### Prediction of the interaction between miR-449b-5p and PSMG3-AS1

IntaRNA 2.0 (http://rna.informatik.uni-freiburg.de/IntaRNA/Input.jsp) was used to predict the interaction between miR-449b-5p and PSMG3-AS1. The sequence of miR-449b-5p was input as short sequence and PSMG3-AS1 sequence was long sequence. All other parameters were set as default.

### RNA preparations

RNAzol (Sigma-Aldrich) was used to isolate total RNA from tissue samples and in vitro cultivated cells. To harvest miRNAs, RNA precipitation and washing were performed using 85% ethanol. The gDNA eraser (Takara Bio) was used to remove genomic DNA from all RNA samples. NanoDrop 2000 Spectrophotometer (Thermo Scientific) was used to measure RNA concentrations. RNA integrity was checked by Urea-PAGE gel.

### RT-qPCR

Precision nanoScript2 Reverse Transcription Kit (Primerdesign) was used to reverse transcribe total RNA samples into cDNA. With cDNA as template, qPCR reactions were prepared using SingleShot™ SYBR® Green Kit (Bio-Rad). The expression levels of PSMG3-AS1 were measured with GAPDH as endogenous control. To measure the expression levels of mature miR-449b-5p, All-in-One miRNA qRT-PCR Detection Kit (GeneCopoeia) was used to complete all steps including polyadenylation, reverse transcription and qPCR assays. U6 was used as the endogenous control of miR-449b-5p. PCR reactions were repeated 3 times and 2^-ΔΔCt^ method was used to normalize gene expression levels. Primer sequences used were as following: 5′-GAAGCAGAACCAACGCACAG-3′ (forward) and 5′-GCATAATCCAATCCCTCAAGAA-3′ (reverse) for PSMG3-AS1; 5′-GTCTCCTCTGACTTCAACAGCG-3′ (forward) and 5′-ACCACCCTGTTGCTGTAGCCAA-3′ (reverse) for GAPDH. Forward primer of miR-449b-5p was 5′-AGGCAGTGTATTGTTAGCT-3′. U6 forward primer and the universal reverse primer were from the kit.

### Cell counting Kit-8 (CCK-8) assay

H522 and H23 cells harvested at 48 h post-transfection were subjected to cell proliferation analysis using a CCK-8 kit (Dojindo). Each well of a 96-well cell culture plate was filled with 5000 cells in 0.1 ml medium. Cells were cultivated under aforementioned conditions and OD values were measured every 24 h for a total of 4 d. CCK-8 solution was added into each well at 4 h before the measurement of OD values.

### Statistical analysis

Mean ± SEM values were used to express data from 3 independent replicates. All statistical analyses were performed using GraphPad Prism6 software (GraphPad, USA). The comparisons between non-tumor and LUAD tissues were performed by paired t test. Comparisons among multiple groups were performed by ANOVA (one-way) and Tukey test. Linear regression was used for correlation analysis. The 64 patients were divided into high (*n* = 32) and low (*n* = 32) PSMG3-AS1 level groups with the mean value of PSMG3-AS1 expression level in LUAD tissues used as a cutoff value. Survival curves were plotted based on the 5-year follow-up data and compared by log-rank test. Chi-squared test was performed to analyze the relationship between the expression levels of PSMG3-AS1 and patients’ clinical data. *p* < 0.05 was considered as statistically significant.

## Results

### Upregulation of PSMG3-AS1 in LUAD predicted poor survival

**Fig. 1:**
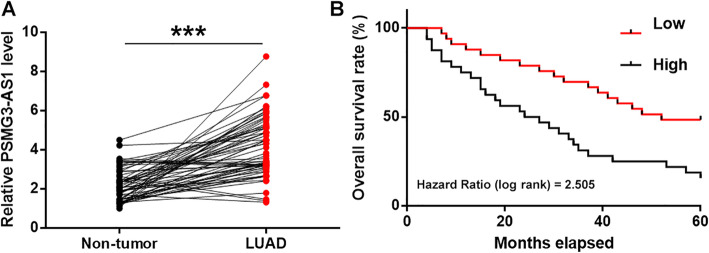
Upregulation of PSMG3-AS1 in LUAD predicted poor survival. Expression levels of PSMG3-AS1 in both LUAD and non-tumor tissues from 64 patients with LUAD were measured by performing RT-qPCR. PCR reactions were repeated 3 times and mean values were presented and compare (**a**). ***, *p* < 0.001. The 64 patients were divided into high and low PSMG3-AS1 level groups (*n* = 32) with the mean value of PSMG3-AS1 expression level in LUAD tissues as a cutoff value. Survival curves were plotted and compared by log-rank test (**b**)

The expression levels of PSMG3-AS1 in both LUAD and non-tumor tissues from the 64 patients with LUAD were measured by RT-qPCR. Compared to non-tumor tissues, the expression levels of PSMG3-AS1 were significantly higher in LUAD tissues (Fig. 1a, *p* < 0.05). Survival curves for both high and low PSMG3-AS1 level groups were plotted. No significant differences in therapeutic treatments were found between high and low PSMG3-AS1 level groups. Compared to the low PSMG3-AS1 level group, patients in the high PSMG3-AS1 level group showed higher mortality rate within 5-year follow-up. Chi-squared test analysis showed that the expression levels of PSMG3-AS1 were not significantly correlated with patients’ age, gender, clinical stages and smoking habit (Table 1).

**Table 1:** Chi-squared test analysis of the correlations between expression levels of PSMG3-AS1 and patients’ clinical data

Items	Groups	Cases	High-expression (n = 32)	Low-expression (*n* = 32)	χ^2^	*p* value
Age (years)	> = 55	33	14	19	1.56	0.21
	< 55	31	18	13		
Gender	Male	42	22	20	0.28	0.60
	Female	22	10	12		
AJCC	I	8	4	4	1.19	0.76
	II	11	6	5		
	III	21	12	9		
	IV	24	10	14		
Smoking	Yes	51	24	27	0.87	0.35
	No	13	8	5		

### MiR-449b-5p is downregulated in LUAD and inversely correlated with PSMG3-AS1

**Fig. 2:**
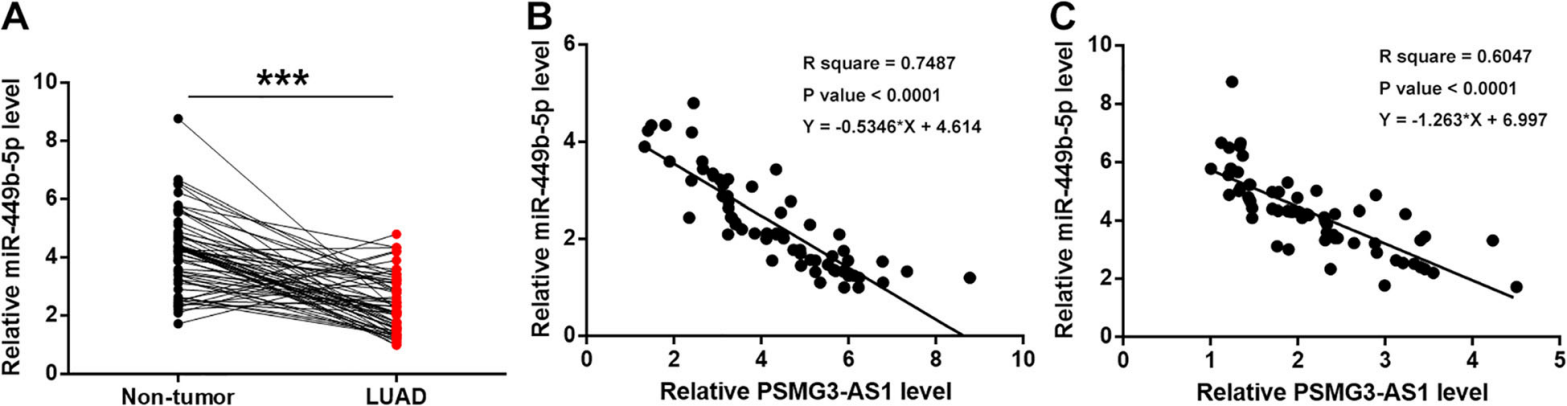
MiR-449b-5p is downregulated in LUAD and inversely correlated with PSMG3-AS1. Expression levels of miR-449b-5p in both LUAD and non-tumor tissues from the 64 patients with LUAD were also measured by performing RT-qPCR. PCR reactions were repeated 3 times and mean values were presented and compare (**a**). ***, *p* < 0.001. Correlations between expression levels of miR-449b-5p and PSMG3-AS1 across both LUAD (**b**) and non-tumor (**c**) tissues were analyzed by linear regression

The expression levels of miR-449b-5p in both LUAD and non-tumor tissues from the 64 patients with LUAD were also measured by RT-qPCR. Compared to non-tumor tissues, the expression levels of miR-449b-5p were significantly lower in LUAD tissues (Fig. 2a, *p* < 0.001). Correlation analysis showed that the expression levels of miR-449b-5p and PSMG3-AS1 were inversely and significantly correlated across both LUAD (Fig. 2b) and non-tumor (Fig. 2c) tissues.

### MiR-449b-5p targeted PSMG3-AS1 to downregulate its expression

**Fig. 3:**
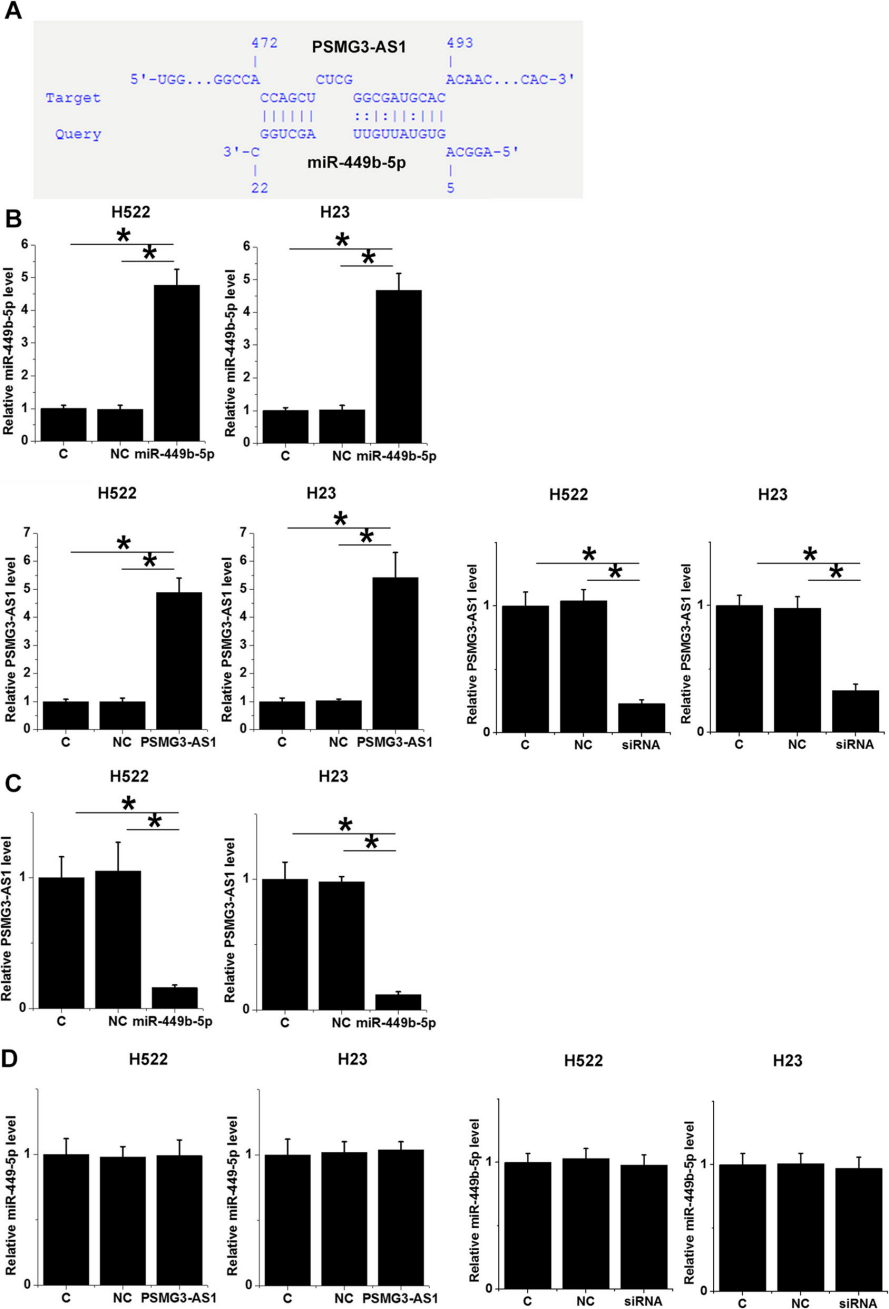
MiR-449b-5p targeted PSMG3-AS1 to downregulate its expression. The interaction between miR-449b-5p and PSMG3-AS1 was predicted using IntaRNA 2.0 (**a**). H522 and H23 cells were transfected with miR-449b-5p mimic, PSMG3-AS1 expression vector, or PSMG3-AS1, followed by the confirmation of miR-449b-5p and PSMG3-AS1 overexpression as well as PSMG3-AS1 siRNA silencing by RT-qPCR at 48 h post-transfection (**b**). The effects of overexpression of miR-449b-5p on PSMG3-AS1 (**c**) and the effects of overexpression of and silencing of PSMG3-AS1 on miR-449b-5p (**d**) were also analyzed by RT-qPCR at 48 h post-transfection. All experiments were repeated 3 times and mean values were presented and compared. *, *p* < 0.05

The interaction between miR-449b-5p and PSMG3-AS1 was predicted using IntaRNA 2.0 [15]. It was observed that miR-449b-5p and PSMG3-AS1 could form multiple bases pairing (Fig. 3a). H522 and H23 cells were transfected with miR-449b-5p mimic, PSMG3-AS1 expression vector, or PSMG3-AS1 siRNA. Overexpression of miR-449b-5p and PSMG3-AS1, and the silencing of PSMG3-AS1 were confirmed by RT-qPCR at 48 h post-transfection (Fig. 3b, *p* < 0.05). Compared to the C and NC groups, overexpression of miR-449b-5p resulted in downregulation of PSMG3-AS1 in cells of both cell lines (Fig. 3c, *p* < 0.05). In contrast, overexpression of and silencing of PSMG3-AS1 did not affect the expression of miR-449b-5p (Fig. 3d).

### MiR-449b-5p targeted PSMG3-AS1 to suppress cancer cell proliferation

**Fig. 4:**
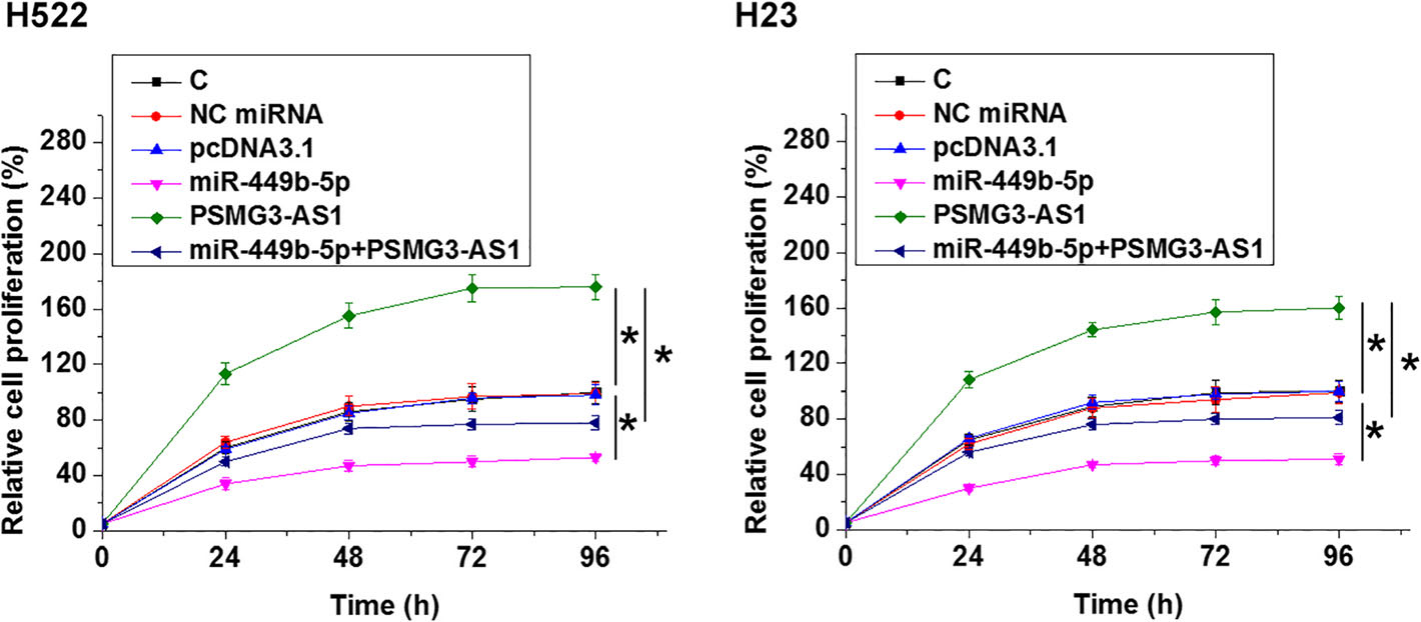
MiR-449b-5p targeted PSMG3-AS1 to suppress cancer cell proliferation. The effects of overexpression of miR-449b-5p and PSMG3-AS1 on the proliferation of H522 and H23 cells were analyzed by performing CCK-8 assay. All experiments were repeated 3 times and mean values were presented and compared. *, *p* < 0.05

**Fig. 5:**
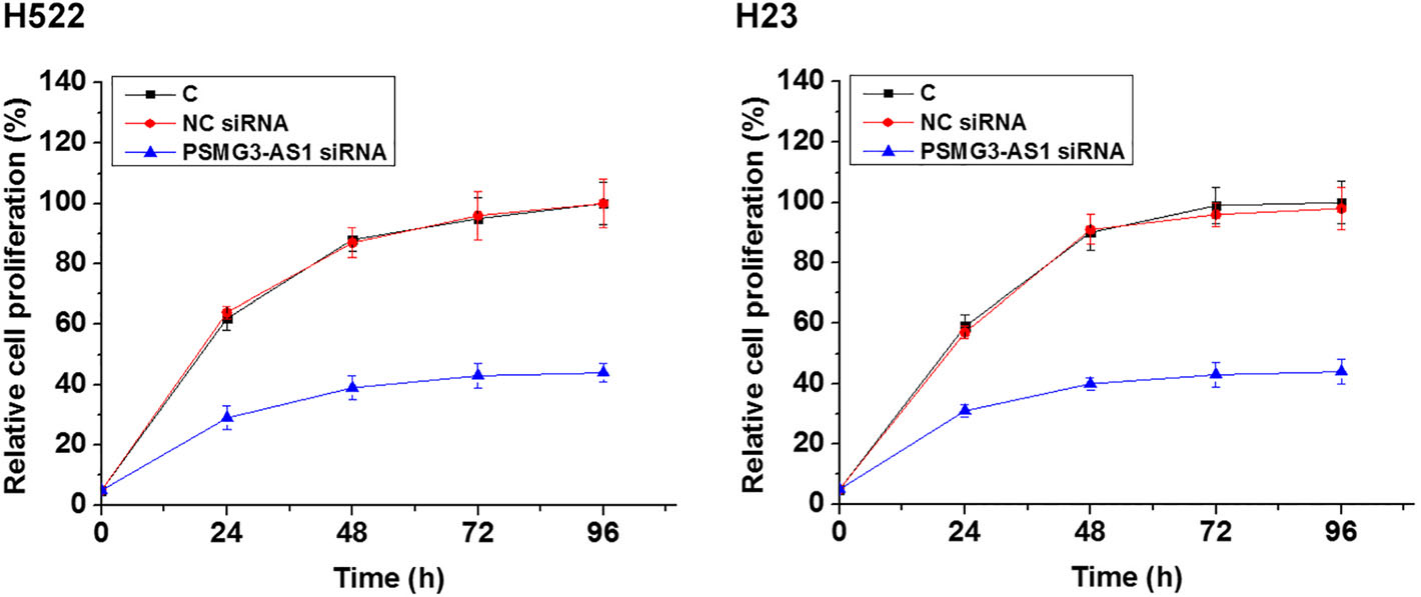
PSMG3-AS1 siRNA silencing suppressed the proliferation of LUAD cells. The effects of silencing of PSMG3-AS1 on the proliferation of H522 and H23 cells were analyzed by performing CCK-8 assay. All experiments were repeated 3 times and mean values were presented and compared. *, *p* < 0.05

The effects of overexpression of miR-449b-5p and PSMG3-AS1 on the proliferation of H522 and H23 cells were analyzed by performing a CCK-8 assay. Compared to the C group, overexpression of PSMG3-AS1 resulted in an increased rate of cell proliferation. Overexpression of miR-449b-5p played an opposite role and reduced the enhancing effects of PSMG3-AS1 on cell proliferation (Fig. 4, *p* < 0.05). In addition, silencing of PSMG3-AS1 was also performed to further confirm the function of PSMG3-AS1 in regulating cell proliferation. Compared to the C group, silencing of PSMG3-AS1 resulted in a decreased proliferation of H522 and H23 cells (Fig. 5, *p* < 0.05).

## Discussion

This study mainly investigated the interaction between miR-449b-5p and PSMG3-AS1 in LUAD. We found that the expression of miR-449b-5p and PSMG3-AS1 were altered in LUAD. In addition, miR-449b-5p might be able to target PSMG3-AS1 to suppress cancer cell proliferation.

The functionality of PSMG3-AS1 has only been investigated in breast cancer [13]. It is observed that PSMG3-AS1 is overexpressed in breast cancer and may promote the migration and proliferation of cancer cells by sponging miR-143-3p [13]. To the best of our knowledge, this study is the first to report the upregulation of PSMG3-AS1 in LUAD, a major subtype of lung cancer. Our in vitro cell experiments also showed that PSMG3-AS1 could promote the proliferation of LUAD cells. Therefore, our data suggested that PSMG3-AS1 played an oncogenic role in LUAD.

Based on our knowledge, the prognostic value of PSMG3-AS1 in cancers remains unknown. Our 5-year follow-up study showed that high expression levels of PSMG3-AS1 measured before therapy were closely correlated with the poor survival of LUAD patients. Most lung cancer patients are diagnosed at advanced stages and the survival is generally poor [16]. Due the lack of early diagnostic markers, the low early diagnostic rate of lung cancer is unlikely to be increased in the near future. Therefore, as an alternative approach, accurate prognosis might help to determine therapeutic approaches and the development of personalized care program, thereby improving the overall survival.

MiR-449b-5p has been characterized as a tumor suppressive miRNA in multiple cancers, such as breast cancer and osteosarcoma [14, 17]. In these cancers, miR-449b-5p targets multiple protein-coding genes, such as CREPT and c-Met, to suppress cancer development and progression [14, 17]. This study is the first to show the downregulation of miR-449b-5p in LUAD and its inhibitory effects on LUAD cell proliferation. Therefore, miR-449b-5p is also a tumor suppressive miRNA in LUAD. Interesting, we proved that miR-449b-5p could target PSMG3-AS1. Therefore, besides protein-coding genes, miR-449b-5p can also target lncRNA to participate in cancer biology.

## Conclusions

In conclusion, miR-449b-5p is downregulated in LUAD and PSMG3-AS1 is upregulated in LUAD. In addition, miR-449b-5p can target PSMG3-AS1 to suppress cancer cell proliferation.

## Data Availability

The datasets used and/or analyzed during the current study are available from the corresponding author on reasonable request.

## Abbreviations

LUAD: Lung adenocarcinoma
lncRNAs: Long non-coding RNAs
ncRNAs: Non-coding RNAs
